# Immunogenicity of a protective intradermal DNA vaccine against lassa virus in cynomolgus macaques

**DOI:** 10.1080/21645515.2019.1616499

**Published:** 2019-06-19

**Authors:** Jingjing Jiang, Preeti Banglore, Kathleen A. Cashman, Connie S. Schmaljohn, Katherine Schultheis, Holly Pugh, Jacklyn Nguyen, Laurent M. Humeau, Kate E. Broderick, Stephanie J. Ramos

**Affiliations:** a Research & Development, Inovio Pharmaceuticals Inc, Plymouth Meeting, PA, USA; b Virology Division, United States Army Medical Research Institute of Infectious Diseases, Fort Detrick, MD, USA; c Office of the Chief Scientists, U.S. Army Medical Research Institute of Infectious Diseases, Fort Detrick, MD, USA

**Keywords:** Immunogenicity, DNA vaccine, lassa virus, intradermal, electroporation

## Abstract

Lassa virus (LASV) is a hemorrhagic fever virus of the Arenaviridae family with high rates of mortality and co-morbidities, including chronic seizures and permanent bilateral or unilateral deafness. LASV is endemic in West Africa and Lassa fever accounts for 10–16% of hospitalizations annually in parts of Sierra Leone and Liberia according to the CDC. An ongoing outbreak in Nigeria has resulted in 144 deaths in 568 cases confirmed as LASV as of November 2018, with many more suspected, highlighting the urgent need for a vaccine to prevent this severe disease. We previously reported on a DNA vaccine encoding a codon-optimized LASV glycoprotein precursor gene, pLASV-GPC, which completely protects Guinea pigs and nonhuman primates (NHPs) against viremia, clinical disease, and death following lethal LASV challenge. Herein we report on the immunogenicity profile of the LASV DNA vaccine in protected NHPs. Antigen-specific binding antibodies were generated in 100% (6/6) NHPs after two immunizations with pLASV-GPC. These antibodies bound predominantly to the assembled LASV glycoprotein complex and had robust neutralizing activity in a pseudovirus assay. pLASV-GPC DNA-immunized NHPs (5/6) also developed T cell responses as measured by IFNγ ELISpot assay. These results revealed that the pLASV-GPC DNA vaccine is capable of generating functional, LASV-specific T cell and antibody responses, and the assays developed in this study will provide a framework to identify correlates of protection and characterize immune responses in future clinical trials.

## Introduction

Lassa virus (LASV) is an Old World Arenavirus and the known cause of hemorrhagic Lassa fever.^1^ It is endemic in West Africa with an estimated 100,000 to 300,000 cases of Lassa fever occurring each year and is classified as a Category A pathogen with pandemic potential by the CDC.^2^ LASV is transmitted via ingestion or inhalation exposure to excretions by its primary reservoir, the *Mastomys* rat, as well as through person-to-person transmission following exposure to blood, tissue, secretions, or excretions of LASV-infected individuals.^3^ Imported cases of Lassa fever have been reported in the United States^4^ and Europe,^5^ and nosocomial infections have occurred in nearly every recorded outbreak.^6^ The clinical symptoms of Lassa infection include fever, malaise, severe edema, blood loss, and acute hemorrhagic fever which are associated with a high mortality rate. The case fatality rate during the most recent 2018 outbreak in Nigeria was 20–30%.^7^ Hearing loss is reported in 1/3 of hospitalized patients with ~50% developing permanent deafness.^8^ Development of an effective vaccine and vaccine delivery technologies to protect those living in Lassa endemic areas are of extreme importance and could prevent this deadly infectious disease from becoming a global health emergency.

Currently there are no FDA-approved preventive vaccines or therapeutics for Lassa fever. Several preclinical LASV vaccine candidates with varying degrees of protection in nonhuman primate (NHP) models of LASV infection have been reported in the literature, and these vaccines have generally been based on recombinant, replication-competent viral vector platforms such as VSV-LASV, YF17D/LASV, and MOPV/LASV.^9–11^ A DNA vaccine approach has the potential to address many challenges with viral vectored approaches in that they do not generate anti-vector responses allowing for repeated boosting, and they are non-replicating and non-integrating.^12^ Unlike conventional inactivated or subunit vaccines, DNA-based vaccines produce robust immune responses that are both cellular and humoral in nature due to their mimicry of natural infection pathways.^13–16^ A DNA-based vaccine also allows for rapid vaccine design and sequence optimization, and cGMP-level production can be rapidly scaled up using relatively simple, low-cost manufacturing methods.^17^ In addition to these platform advantages, Inovio has developed a thermostable DNA vaccine formulation allowing for greater than four years stability at 2–8°C (refrigerated, non-frozen) and one year stability at room temperature (Ramos et al, manuscript in preparation).

We developed a codon-optimized DNA vaccine encoding the LASV glycoprotein complex (GPC) and previously reported on its ability to provide complete protection against death, clinical signs of illness (including deafness) and viremia in Guinea pig and NHP models of lethal Lassa infection.^18,19^ In the current study we report on the immunogenicity of the LASV DNA vaccine in NHPs and provide evidence of its ability to induce both antibody and cellular responses that may contribute to protection against lethal LASV challenge.

## Results

### Expression of LASV GPC antigen following intradermal electroporation delivery

**Figure 1.**
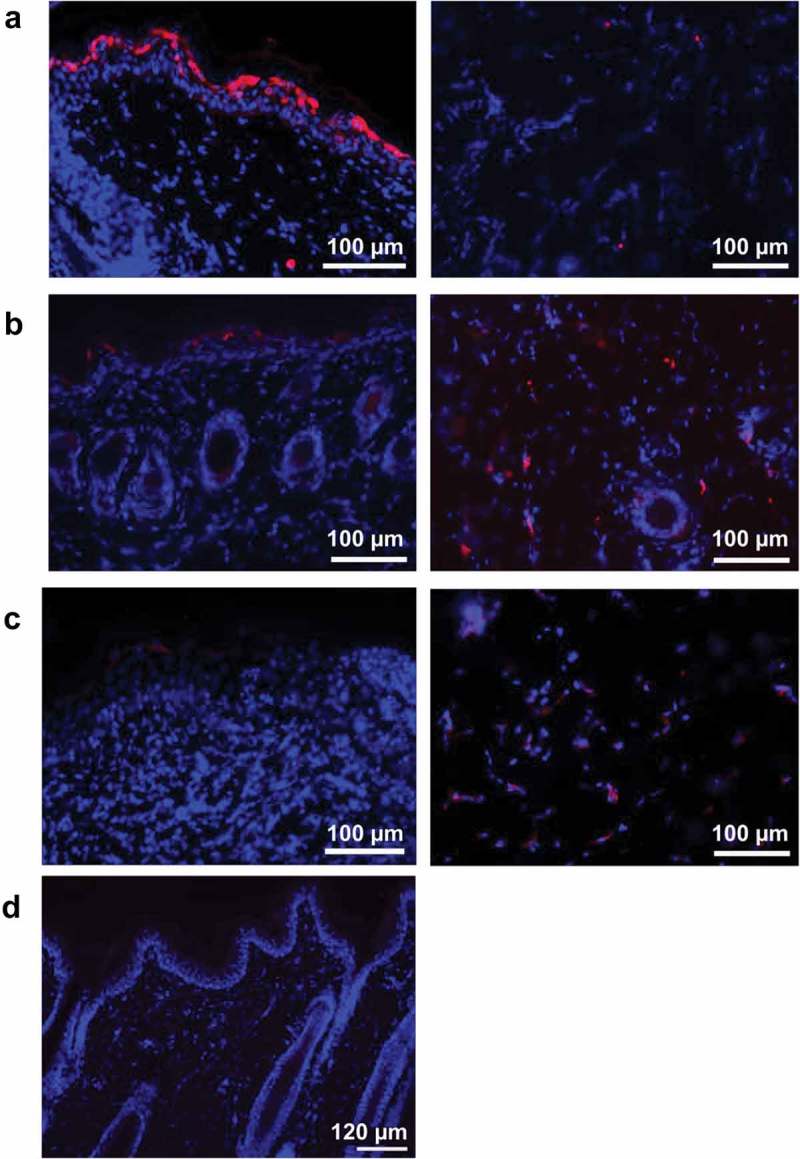
Intradermal pLASV-GPC vaccine antigen expression following ID-EP delivery. Guinea pigs were immunized intradermally with pLASV-GPC DNA vaccine (a–c) or empty vector (d) by ID-EP delivery. Skin tissue samples were collected at 24 (a,d), 48 (b), or 72 (c) hours post-immunization for antigen expression (red) and epidermis (left panel) and dermis (right panel) were imaged by fluorescent microscopy. Red indicates LASV GPC staining and blue indicates Hoechst counterstaining of nuclei.

We have previously reported on *in vivo* intradermal electroporation (ID-EP) delivery of plasmid DNA resulting in robust plasmid gene expression within treated skin,^20^ and that ID-EP delivery of a plasmid DNA vaccine encoding LASV GPC antigen results in 100% protective immunity against lethal LASV challenge to Guinea pigs and NHPs.^18,19^ Here we evaluated the *in vivo* expression pattern of DNA vaccine-encoded LASV GPC antigen following ID-EP delivery in Guinea pigs. Guinea pigs were immunized with pLASV-GPC DNA vaccine by ID-EP and skin samples were collected at 24, 48, and 72 h post-treatment to measure antigen expression by immunofluorescence microscopy. At 24 h post-treatment, directly transfected cells expressing LASV GPC appeared in the epidermal sections of skin with scattered transfection in the dermal layer (Figure 1(a)). At 48 h post-treatment, the number of transfected keratinocytes observed in the epidermis layer decreased as a result of desquamation, while LASV GPC expression became visible in the dermal layer due to migration of transfected cells (Figure 1(b)). By 72 h post-treatment, increased cell infiltration and LASV GPC expression were observed throughout the dermal region (Figure 1(c)). We previously demonstrated the direct transfection of both keratinocytes and dendritic cells in the epidermis with the ID-EP device used to deliver the pLASV-GPC DNA vaccine,^20,21^ suggesting the same populations of cells were targeted by the vaccine in the current study.

### Protective efficacy of pLASV-GPC DNA vaccine in NHPs

**Figure 2.**
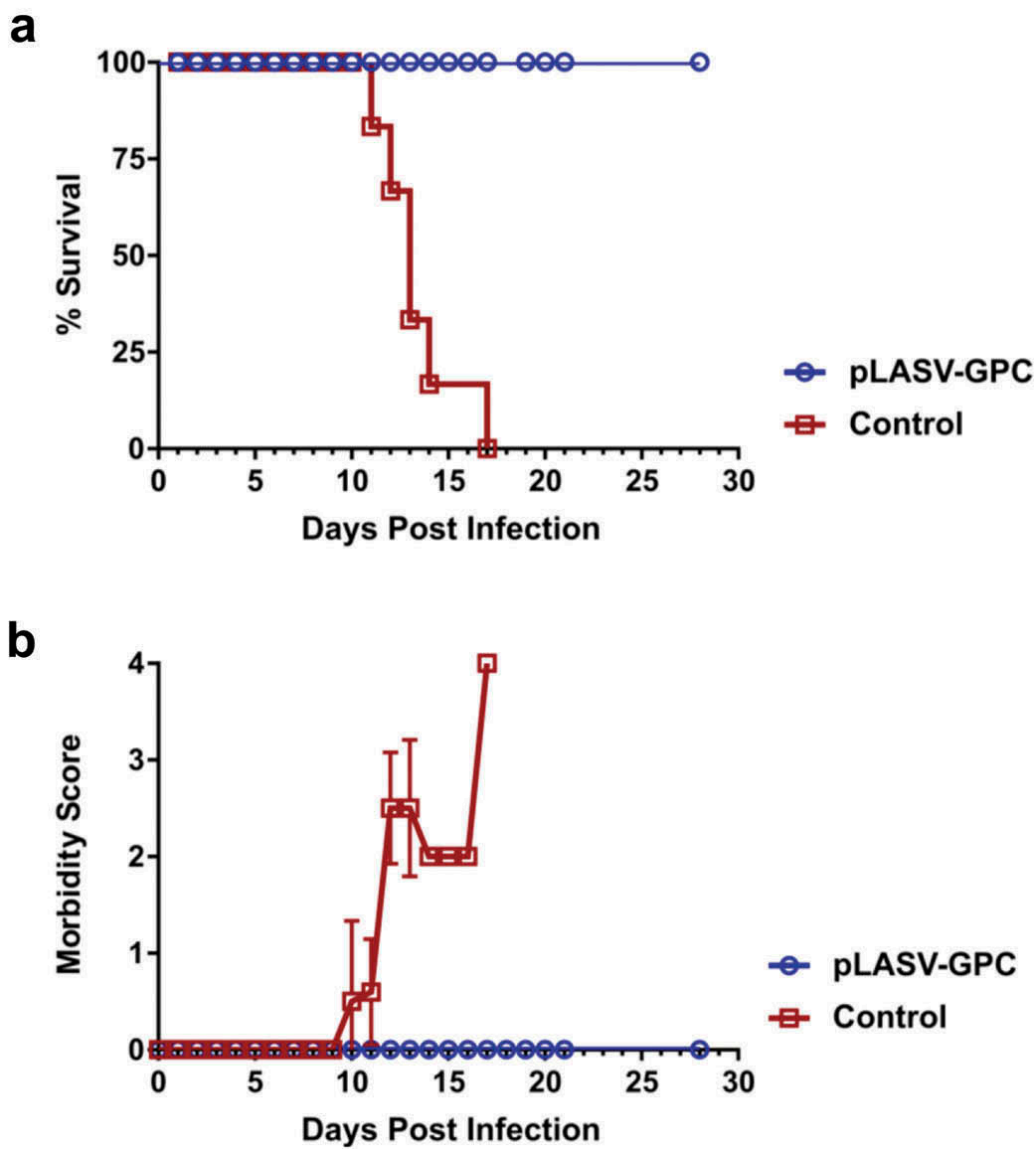
Protective efficacy of pLASV-GPC vaccine against lethal viral challenge in NHPs. pLASV-GPC DNA-immunized and non-immunized control NHPs (n of 6 each) were challenged with a lethal dose of LASV at four weeks post-final immunization. NHPs were monitored daily for survival (a) and clinical signs of disease (b).

The Cynomolgus macaque model of LASV infection closely mimics the clinical signs of disease and outcomes observed in humans infected with LASV. NHPs develop severe hemorrhagic disease and most animals become moribund or die between 11 and 18 days post-LASV infection, and NHPs that survive the hemorrhagic phase of disease develop neurological disorders including tremors, ataxia, and seizures requiring their euthanasia, with few NHPs recovering fully from disease.^22^ NHPs that survive infection without experiencing severe signs nor full recovery develop unilateral or bilateral deafness,^23^ a known outcome of LASV infection in approximately 30% of surviving human patients. We previously demonstrated the ability of the pLASV-GPC DNA vaccine to induce protective immunity against lethal LASV challenge and associated illness in NHPs.^19^ In an effort to characterize vaccine-induced immune responses, we repeated this study so that pre-challenge samples could be collected and assessed in various immunoassays. Cynomolgus macaques of Mauritius origin (n of 6) received two immunizations spaced four weeks apart by ID-EP delivery of 2 mg pLASV-GPC. Non-immunized cynomolgus macaques (n of 6) served as infection controls. At four weeks post final immunization, NHPs were challenged i.m. with a target dose of 1,000 pfu LASV and subsequently monitored daily for survival and clinical signs of disease. In control animals, onset of clinical signs of disease occurred between days 10 to 12 post-exposure (Figure 2(b)), and all control NHPs either met criteria for euthanasia or succumbed to Lassa fever between days 11 to 17 post-exposure (Figure 2(a)). In contrast, all immunized NHPs survived LASV infection with no clinical signs of disease (Figure 2(a,b)), replicating the results of earlier studies.^19^

### Humoral immune responses in protected NHPs

**Figure 3.**
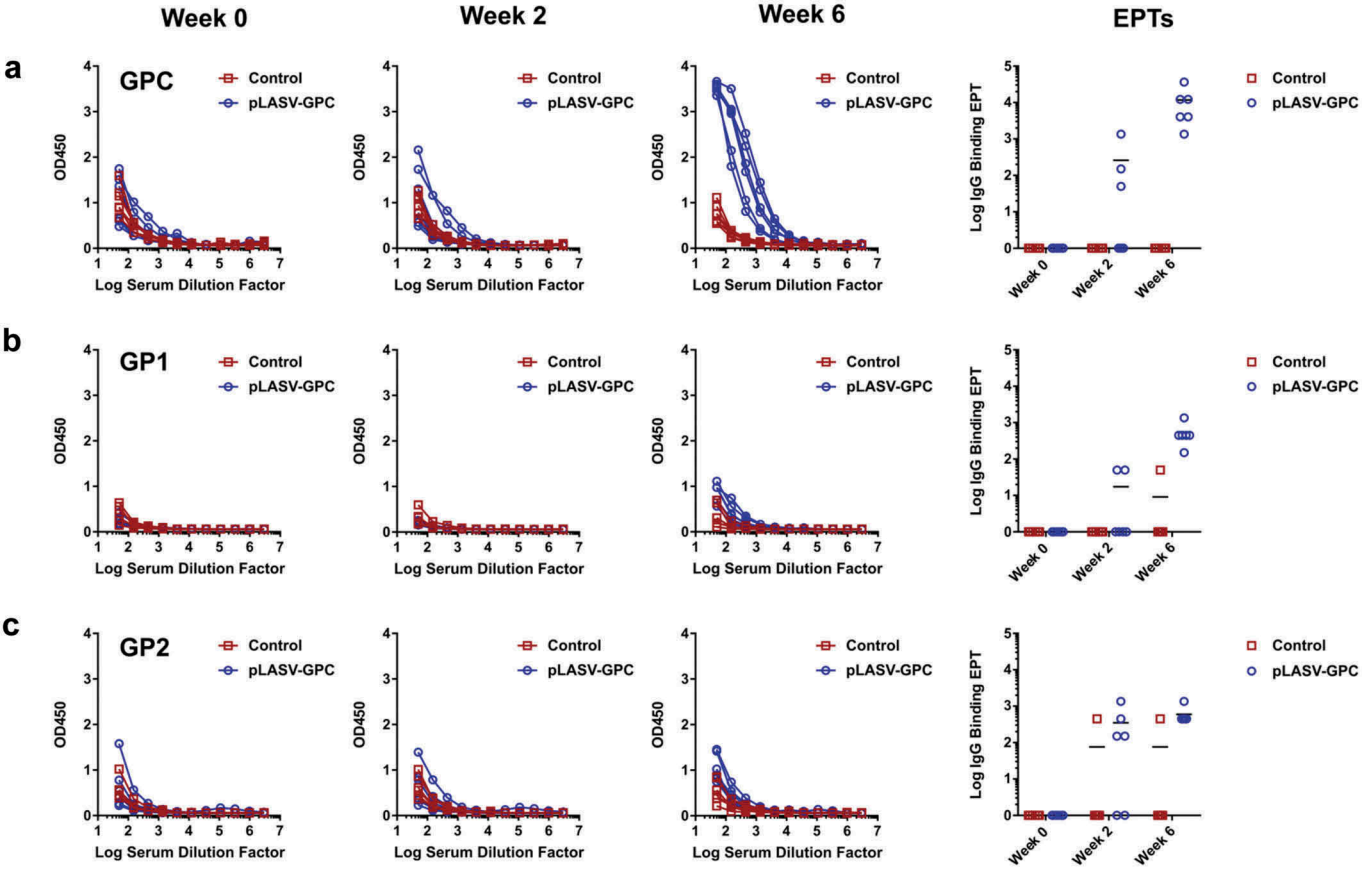
Pre-challenge LASV-specific binding antibodies. NHP serum samples were collected at Weeks 0, 2, and 6 post-immunization and before challenge as described in Figure 2. Specific IgG binding against LASV GPC (a), GP1 (b), and GP2 (c) were measured by ELISA and endpoint titers (EPTs, right panel) were calculated as described in the methods.

The LASV GPC is composed of GP1 and GP2 subunits. Antibodies isolated from memory B cells of human Lassa fever survivors have been reported to bind either GP1, GP2, or the assembled GPC.^24^ We therefore assessed pre-challenge humoral responses to each of these potential antigens in pLASV-GPC DNA-immunized NHPs that were protected from lethal LASV challenge. Antibodies against LASV-GP1, GP2, and GPC were measured by binding IgG ELISAs in sera samples collected before and two weeks after each immunization. Binding antibodies to assembled GPC were detected in 3 of 6 NHPs after single immunization, with 100% seroconversion after two immunizations with a mean endpoint titer of 11,700 (4,050 to 36,450; Figure 3(a)). Endpoint binding titers to GP1 and GP2 were much lower than those observed for GPC with mean endpoint titers of 550 (150 to 1,350) and 600 (450 to 1,350), respectively (Figure 3(b,c)). There were no binding antibodies to GP1, GP2, or GPC in control NHPs (Figure 3(a–c)).

**Figure 4.**
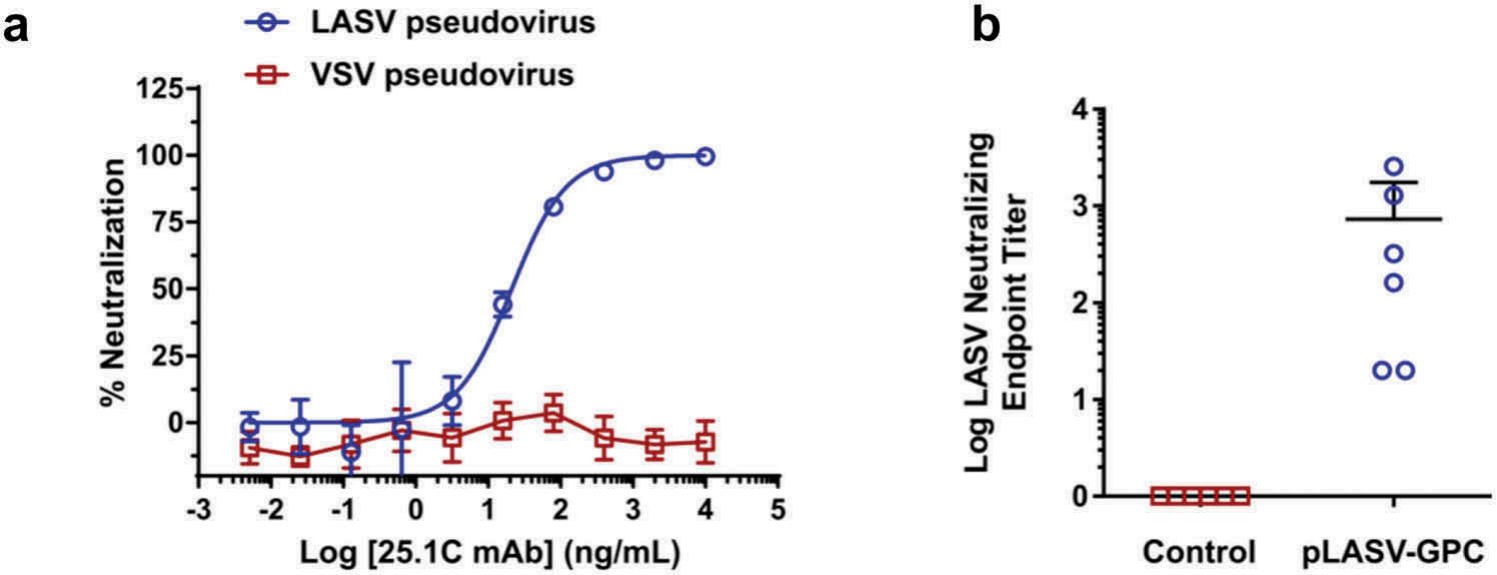
Pre-challenge LASV-specific neutralizing antibodies. (a) Specific, dose-dependent neutralization of LASV, but not VSV, pseudovirus by LASV GPC-specific monoclonal antibody 25.1C. (b) Pre-challenge NHP serum samples collected at weeks 0 and 6 post-immunization were measured for LASV pseudovirus neutralizing activity and neutralizing EPTs calculated as described in the methods.

It has been reported that most LASV neutralizing antibodies from infected survivors bind assembled GPC and not GP1 or GP2.^24^ We therefore developed a LASV pseudovirus assay to allow measurement of serum neutralizing activity in a BSL-2 lab. As shown in Figure 4(a), this assay is specific for LASV as anti-LASV-GPC mAb 25.1C neutralized pseudovirus displaying LASV-GPC, but not pseudovirus displaying irrelevant GP, with an IC50 of 11 ng/mL comparable to a previously published report.^24^ Neutralizing activity was measured in NHP sera samples collected at week 0 and two weeks after second immunization. Sera from all vaccinated animals demonstrated neutralizing activity against LASV pseudovirus with a mean titer of 727 (20 to 2,560), while sera from control NHPs had no detectable neutralizing activity (Figure 4(b)).

### Cellular immune responses in protected NHPs

**Figure 5.**
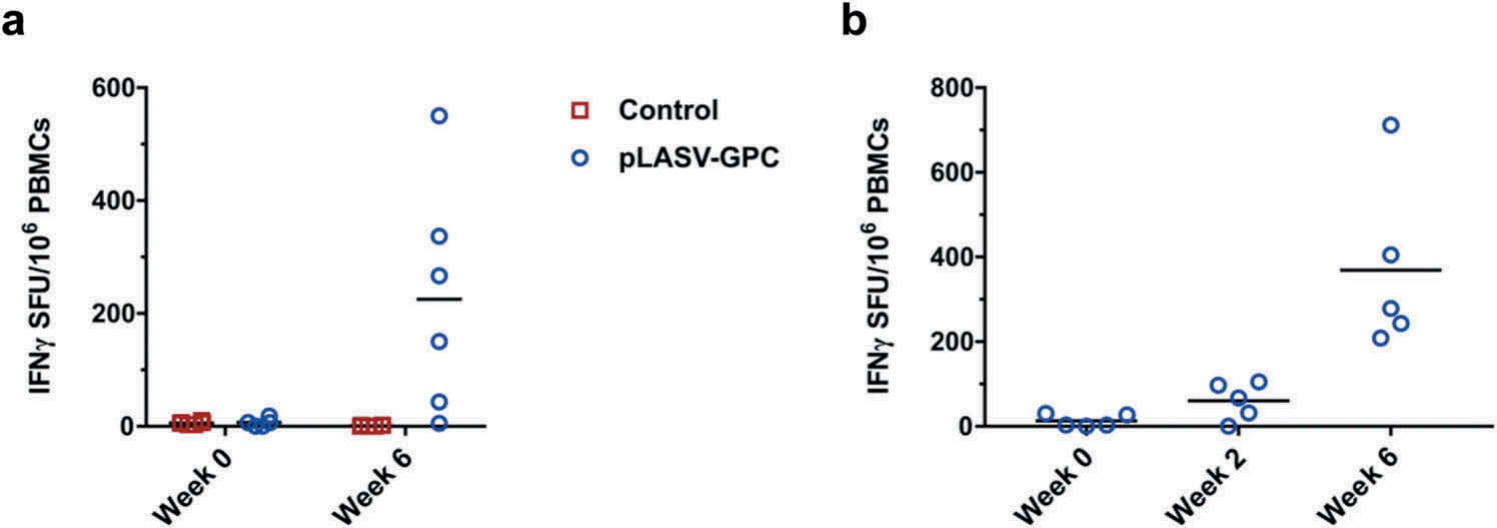
Pre-challenge LASV-specific cellular responses. Specific cellular responses to LASV GPC peptides were measured by IFNγ ELISpot as described in the methods. (A) Pre-challenge LASV-specific cellular responses at the indicated timepoints post-immunization for control and pLASV-GPC DNA-immunized NHPs described in Figure 2. (B) LASV-specific cellular responses at the indicated timepoints post pLASV-GPC immunization for NHPs in a follow-up study with the same immunization protocol as (A).

It has been previously proposed that T cells play a role in protection against lethal Lassa fever.^25^ We therefore evaluated pre-challenge cellular responses in pLASV-GPC DNA-immunized NHPs that were protected from lethal LASV challenge. T cell responses were measured in NHPs at week 0 and two weeks post second immunization by IFNγ ELISpot after PBMC stimulation with LASV GPC peptides. LASV GPC specific T cell responses were detected in five of six protected NHPs following pLASV-GPC immunization, but not in control NHPs (Figure 5(a)). During the course of the study it was noted that blood samples collected at the six week timepoint were not processed until two days after draw due to sample shipping delays. It is known that prolonged time between blood draw and PBMC isolation can negatively impact PBMC viability and functionality.^26^ We therefore immunized a second cohort of NHPs (*n* = 5) under the same conditions as those used in the challenge study, while collecting and processing blood samples under industry-standard conditions for use in IFNγ ELISpot assays. In this second study, LASV GPC T cell responses were detected in three of five NHPs after first immunization and five of five after second immunization (Figure 5(b)) at levels greater than those observed in the first study. Combined with the ELISA and pseudovirus neutralization results, these data confirm that ID-EP delivery of the pLASV-GPC DNA vaccine can generate functional antibody and T cell responses to Lassa virus in NHPs.

## Discussion

Currently there are no defined correlates of protection against Lassa disease to support LASV vaccine development, although data from human Lassa survivors and animal models strongly suggest that cellular immunity is sufficient to confer protection while antibodies may also be protective. Published data in humans suggested early and high cellular responses are associated with LASV clearance while little to no antibodies were observed until late in the convalescent phase of infection (reviewed in Prescott et al., 2017^27^), and data from NHPs that survived LASV challenge also supported a role for cellular immune responses in control of infection.^28^ Also, vaccines that target intracellular LASV nucleoprotein (NP) did offer some degree of protection in the NHP model. However, protection against lethal disease was less than 50% in these studies, and NP-based vaccines did not protect against viremia, suggesting either additional antigen targets or mechanisms beyond T cells are required for complete protection against LASV.^11,25,29^ Although reports suggested limited humoral responses during Lassa infection, neutralizing monoclonal antibodies isolated from human Lassa infection survivors during convalescence were efficacious in Guinea pig and NHP challenge models.^30,31^ LASV vaccines have been reported in the literature, although detailed immunology data in NHP models are limited and do not support definition of a correlate of protection. Anti-LASV antibodies and cytokine-producing cellular responses have been observed in NHPs following immunization with replication-competent, viral vectored vaccines such as the VSV-LASV and MOPV/LASV reassortment ML29 vaccines.^32,33^ Therefore, both cellular and humoral immune responses should be investigated as potential mechanisms of LASV vaccine mediated immunity. Here we demonstrated the ability of an efficacious pLASV-GPC DNA vaccine to generate both cellular and humoral responses in NHPs.

All NHPs immunized with pLASV-GPC developed robust binding antibody titers prior to lethal LASV challenge. pLASV-GPC vaccine-induced antibodies preferentially bound the assembled LASV GPC over GP1 and GP2, binding properties associated with antibody-mediated LASV neutralization in human Lassa survivors^24^ and efficacious monoclonal antibodies against lethal LASV infection in animals.^30,31^ We believe this is the first NHP study characterizing vaccine-induced antibody binding to different LASV GP forms. Previously we reported low-to-no levels of neutralizing antibodies following pLASV-GPC DNA immunization that rapidly increased after viral challenge as measured by live plaque reduction neutralization titer (PRNT) assay, similar to what was reported with VSV-LASV immunization.^19,32^ These results suggested pLASV-GPC DNA vaccination resulted in a B cell population capable of responding rapidly to LASV infection by producing neutralizing antibodies, and that pre-challenge neutralizing antibodies were possibly present at levels below detection in stringent PRNT assays. We therefore developed a LASV pseudovirus assay to allow detection of potential neutralizing activity with greater sensitivity, as was previously published in a functional screen of LASV monoclonal antibodies.^24^ Indeed, we were able to detect pseudovirus neutralizing antibodies in all pLASV-GPC DNA-immunized, protected NHPs before challenge. This BSL-2 assay could provide a safe alternative to live PRNT assays for evaluating neutralizing antibody responses in LASV vaccine clinical trials.

pLASV-GPC DNA vaccination of NHPs generated robust IFNγ-secreting T cells that recognize LASV GPC prior to lethal LASV challenge. In a second study, pLASV-GPC-induced T cell responses were observed as early as two weeks post first immunization in several NHPs, and in all NHPs after two immunizations.^32^ Investigation into additional T cell responses, such as multi-cytokine and cytolytic effector functions, will be performed in future pLASV-GPC DNA vaccine studies.

Lassa virus isolates have a high level of genetic diversity and GPC amino acid identities of different Lassa viral strains can vary by up to 14.8%.^34^ Lassa viruses are categorized into four lineages that can be traced to their geographically endemic regions, with LASV clades I-III localized in Nigeria and clade IV in Sierra Leone, Liberia, and Guinea.^34,35^ The antigen sequence encoded by the pLASV-GPC DNA vaccine matches the GPC amino acid sequence of the clade IV LASV Josiah strain, the same VSV-LASV antigen that was shown to induce cross-protective immunity against LASV viral strains of multiple clades.^11^ It is therefore highly likely the pLASV-GPC DNA vaccine will also protect against multiple LASV strains, which will be important as the LASV lineages in the current Nigerian outbreak have yet to be clearly defined. However, this theory will need to be tested in heterologous viral challenge models.

Given the limited knowledge of requirements for vaccine-mediated protection against Lassa disease, significant effort must be placed on developing methods to monitor a variety of potential anti-LASV immune responses for vaccine development. We have developed multiple methods to measure LASV-specific T cell and antibody responses in NHPs which we used to characterize the immunogenicity of a protective DNA vaccine. Although these studies were too small to define correlates of protection, larger dose-ranging studies are currently being planned that will allow us to define correlates of LASV DNA vaccine protection in NHPs will be translated for future use in clinical trials. The LASV DNA vaccine induced both T cell and antibody responses, both of which likely contribute to vaccine-mediated protection against LASV infection. The findings of this study represent a major contribution to the field of Lassa vaccine development and evaluation and has the very promising potential to be effective for the prevention of Lassa fever, a disease that causes significant human morbidity and mortality.

## Materials and methods

### Construction of LASV GPC vaccine plasmid

The pLASV-GPC DNA plasmid encodes a codon-optimized full-length LASV glycoprotein complex precursor (GPC, Genbank Accession number AY628203.1) from the Josiah strain cloned into pWRG7077 vector as previously described.^19^

### Recombinant LASV glycoproteins

LASV glycoprotein (GP) 1, GP2, and GPC sequences from the Josiah strain (Genbank Accession number AY628203.1) were modified by deletion of transmembrane domains, individually synthesized with 6-His tags, and then subcloned into bacterial expression vectors for E.coli expression and subsequent protein purification by Nickel column (GenScript). Purified proteins were sterile filtered using a 0.22 micron filter and analyzed by SDS-PAGE and Western blot for purity and molecular weight (Supplemental Figure 1), and BCA assay for protein concentration. Peptides consisting 15-mers overlapping by 9 amino acids and spanning the length of the above LASV GPC sequence (GenScript) were reconstituted in DMSO and combined into three peptide pools for PBMC stimulation as outlined in Supplementary Table 1.

### Animals

Female Dunkin-Hartley Guinea pigs (*Cavia porcellus*) aged 8–10 weeks and weighing 500–600g were group housed with ad libitum access to food and water at BioTox Sciences (San Diego, CA). EP procedures and blood collections were performed while Guinea pigs were maintained under general anesthesia by inhaled isoflurane. For terminal studies, Guinea pigs were first placed under general anesthesia by inhaled isoflurane and then humanely euthanized by intracardiac injection of pentobarbital. Mixed male and female Cynomolgus macaques (*Macaca fascicularis* of Mauritius origin) weighing 2.25–6.25 kg were individually housed and acclimated for 4 weeks before experimentation and during the immunization/pre-challenge phase under standard conditions at Bioqual (Rockville, MD) before being transferred to USAMRIID (Fort Detrick, MD) for BSL-4 LASV challenge. All animal research was conducted under IACUC approved protocols through respective institutions (BioTox Sciences, Bioqual, and USAMRIID) in compliance with the Animal Welfare Act, PHS Policy, AALACi guidelines, USDA, and other Federal statutes and regulations relating to animals and experiments involving animals. The BioTox Sciences, Bioqual, and USAMRIID facilities where this research was conducted are accredited by the Association for Assessment and Accreditation of Laboratory Animal Care, International and adhere to principles stated in the Guide for the Care and Use of Laboratory Animals, National Research Council, 2011.

### pLASV-GPC plasmid gene expression in Guinea pig skin

Guinea pigs were shaved and depilated 1 day before treatment. Guinea pigs were then injected with 100 μl of 1 mg/mL pLASV-GPC DNA plasmid by Mantoux method immediately followed by intradermal EP. Guinea pigs were humanely euthanized as described above for collection of treated skin biopsies at 24, 48, and 72 h post treatment for immunofluorescence analyses. Skin biopsies were fixed in 4% paraformaldehyde (Sigma-Aldrich, cat. 1004969010) at 4°C overnight. The next day, skin biopsies were buffered in a 30% sucrose solution (Sigma-Aldrich, cat. ARK2195B) and stored at 4°C. For sectioning biopsies were embedded in O.C.T. compound (Sakura, cat. 4583) and sectioned at a thickness of 15 μm, using an OTF cryostat (Bright Instruments, Cambridge, UK). Sections were stained with unconjugated primary rabbit anti-LASV GP (IBT Biosciences, cat. 0307–001), and then stained with donkey anti-rabbit AF555-conjugated secondary antibody (Life technologies, cat. A-31572). An additional stain, Hoechst 33342 (Life Technologies, cat. H3570), was used to visualize nuclei. The slides were then mounted with Fluoromount (eBioscience, cat. 00–4958-02) and viewed by fluorescence microscopy using an Olympus BX51 with a U-TV1X-2/U-CMAD 3 combo camera for photo acquisition (Olympus, Melville, NY). MagnaFire software was used to acquire the images.

### NHP immunizations and LASV challenge

NHP immunizations: NHPs were physically restrained for anesthesia by pulling the rear cage wall forward so that safe access to the muscles of the lower limbs could be achieved for intramuscular (IM) injection of ketamine anesthesia and/or blood draws. Appropriate mg/kg doses of anesthetic were calculated for each animal based on the last known weight, according to standard operating procedures (SOP). Cynomolgus macaques (n of 6) were injected intradermally by Mantoux method followed by CELLECTRA® ID-EP on weeks 0 and 4 for a total of two immunizations each at a dose of 2 mg pLASV-GPC DNA in 0.2 mL split across 2 treatment sites. Non-immunized, non-injected macaques (n of 6) served as controls. Sera and BD Vacutainer EDTA whole blood samples were collected from the femoral vein on weeks 0, 2 and 6 for ELISAs and weeks 0 and 6 for interferon gamma (IFNγ) enzyme-linked immunospot (ELISpot) and pseudovirus neutralization analyses.

LASV challenge: NHPs were restrained and anesthetized as described for immunization procedures. NHPs were challenged with LASV at 4 weeks after second immunization as previously described.^19^ LASV (Josiah strain) was diluted to a concentration of 1000 pfu/mL in sterile physiological saline and NHPs were given a single IM injection of 1000 pfu LASV. Blood samples were collected from anesthetized primates via venipuncture of the femoral vein. NHPs were monitored daily for disease progression and were euthanized when moribund or at study completion at 28 days post-challenge according to IACUC-approved euthanasia criteria.

Morbidity scoring and euthanasia criteria: Each NHP was evaluated daily and assessed on a morbidity scale of 0–4 depending on disease severity as follows: 0 = alert, responsive, normal activity, free of disease signs or exhibits only resolved/resolving disease signs; 1 – slightly diminished general activity, subdued but responds normally to external stimuli; 2 – withdrawn, may have head down, fetal posture, hunched, reduced response to external stimuli; 3 – prostrate but able to rise if stimulated or moderate to dramatically reduced response to external stimuli; 4 – persistently prostrate and/or observed to have experienced a seizure. NHPs with a score of 4 or a score of 3 with rectal temperature ≤ 34°C met euthanasia criteria. When a primate was assessed to have met euthanasia criteria, they were administered an IM injection of Telazol according to SOP, then were euthanized by barbiturate overdose. Death was confirmed by the absence of a heartbeat for at least 10 min. After samples were collected, animal carcasses were autoclaved using a validated sterilization protocol to be removed from the BSL-4 suite, then were ultimately incinerated as regulated medical waste according to SOP.

### ELISAs

ELISAs were performed to determine sera antibody binding titers. Nunc ELISA plates were coated with 1 µg/ml recombinant LASV GP1, or GP2 or GPC in Dulbecco’s phosphate-buffered saline (DPBS) overnight at 4°C. Plates were washed three times then blocked with 3% bovine serum albumin (BSA) in DPBS with 0.05% Tween 20 for 2 h at 37°C. Plates were then washed and incubated with serial dilutions of NHP sera and incubated for 2 h at 37°C. Plates were again washed and then incubated with 1:10,000 dilution of horse radish peroxidase (HRP) conjugated anti-nonhuman primate IgG secondary antibody (Sigma-Aldrich, cat. A0170) and incubated for 1 h at 37°C. After final wash plates were developed using SureBlue^TM^ TMB 1-Component Peroxidase Substrate (KPL, cat. 52–00-03) and the reaction stopped with TMB Stop Solution (KPL, cat. 50–85-06). Plates were read at 450 nm wavelength within 30 min using a SpectraMax Plus 384 Microplate Reader (Molecular Devices, Sunnyvale, CA). Binding antibody endpoint titers (EPTs) were calculated as previously described.^13^

### PBMC isolation and ELISpot assays

Blood samples were collected in EDTA K2 tubes and peripheral blood mononuclear cells (PBMCs) were isolated using 90% Ficoll gradient centrifugation in SepMate tubes according to the manufacturer’s instructions (Stemcell Technologies, cat. 85460), then washed and resuspended in Roswell Park Memorial Institute (RPMI) 1640 media supplemented with 10% FBS and 1% Penicillin-Streptomycin (R10 media). NHP IFNγ ELISpot assays were performed using commercial Mabtech IFNy ELISpot kits (Mabtech, cat. 3420M-2APW-10). Briefly, 96-well ELISpot plates pre-coated with capture antibody were blocked with R10 media overnight at 4°C. The following day, 200,000 PBMCs in R10 media were added to each well and incubated at 37°C in 5% CO_2_ in the presence of LASV GPC peptide pools, dimethyl sulfoxide (DMSO, negative control), or phorbol myristate acetate plus ionomycin (positive control). After 18–20 h, plates were washed and developed according to the manufacturer’s instructions and IFNγ positive spots were counted using an automated ELISpot reader (CTL, Shaker Heights, OH). Antigen-specific responses were determined by subtracting the number of spots in DMSO-treated from peptide-treated wells. Results are shown for average spot-forming units (SFU)/10^6^ PBMCs obtained for triplicate wells.

### LASV pseudovirus neutralization assay

Pseudovirus production: HIV-based LASV pseudoviruses expressing a luciferase reporter gene were generated by co-transfection of 293T cells with pLASV-GPC plasmid and envelope-defective NL43R-E_LUC (NIH AIDS reagent program) using Lipofectamine 3000 (Life Technologies, cat. L3000075). The pseudovirus-containing culture supernatant was harvested 72 h post transfection and then centrifuged at 500 g for 5 min. Pseudovirus aliquots were stored at −80°C until use in neutralization assays.

Pseudovirus neutralization: NHP sera samples were heat inactivated for 30 min at 56°C prior to testing. Serial dilutions of heat inactivated sera were mixed with equal volumes of pseudovirus in DMEM supplemented with 10% FBS and 1% penicillin-streptomycin (D10) and incubated 1 h at 37°C, then added to 293T cells that were seeded one day prior in 96-well cell culture plates. Following 72 h of incubation in 5% CO2 at 37°C, luciferase signal was quantified by Bright-Glo Luciferase Assay System (Promega, cat. E2650) according to manufacturer’s instructions and the luminescence (RLU) was read with Spectra Max HTS plate reader (BioTek, Winooski, VT). Neutralizing antibody was measured by the reduction in luciferase signal in sera comparison to an infection control. For each animal, neutralization titer was the maximum sera dilution with significant RLU value over day 0 background as determined by two-way ANOVA. Neutralizing antibody specificities were confirmed using monoclonal antibodies against LASV (clone 25.1C, Zalgen Labs, cat. Ab02510C).

### Statistical analysis

GraphPad Prism 7.02 was used to analyze data. Statistical differences in binding antibody or neutralization EPTs between control and treatment groups at each timepoint were determined by Wilcoxon signed-rank test or Mann-Whitney test. Statistical differences in ELISpot SFUs between control and treatment groups were assessed by parametric t-test, or between pre-immunization and post-immunization timepoints by two-way ANOVA. Values of *p* < .05 were considered statistically significant.
