# Supplementary material for: Immunogenicity of a protective intradermal DNA vaccine against lassa virus in cynomolgus macaques
Source: Hum Vaccin Immunother. 2019 Jun 19;15(9):2066–74. doi: 10.1080/21645515.2019.1616499 (PMC6773375; doi:10.1080/21645515.2019.1616499)
Supplement: Supplemental Material [file khvi-15-09-1616499-s001.docx]

**Supplementary Table S1: LASV GPC peptide sequences and pooling strategy.**

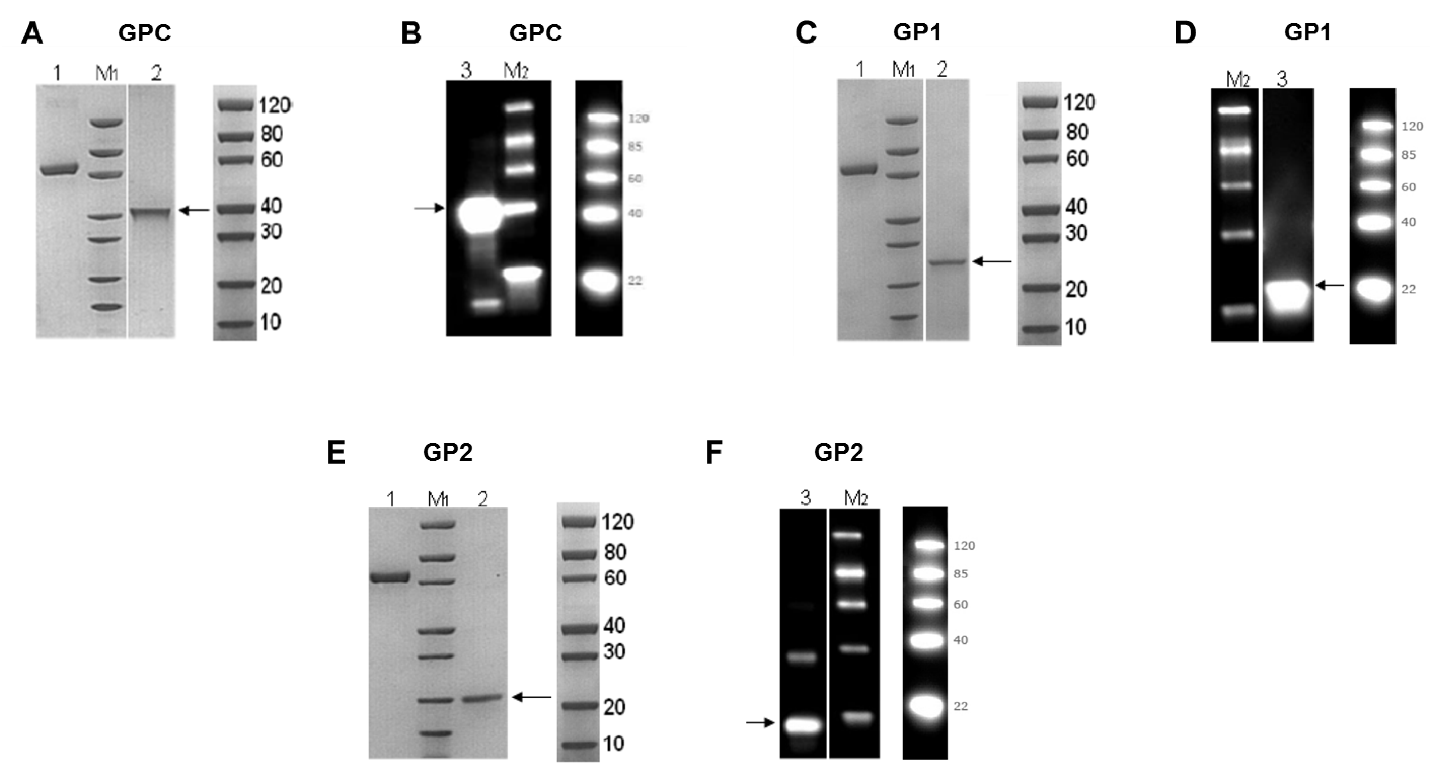


**Supplementary Figure S1: Analysis of purified LASV GPC, GP1 and GP2 proteins.** Proteins were expressed and purified as described in the Materials and Methods and their purities and identities confirmed by coomassie staining (A-C) and anti-His Western blot (D-E) after resolution by SDS-PAGE. Lane 1: 2 µg BSA; Lanes 2 and 3: 2 µg LASV GPC (A), GP1 (B) or GP2 (C) proteins; Lane M1: protein marker (GenScript, Cat. No. M00516); Lane M2: protein marker (GenScript, Cat. No. MM0908)


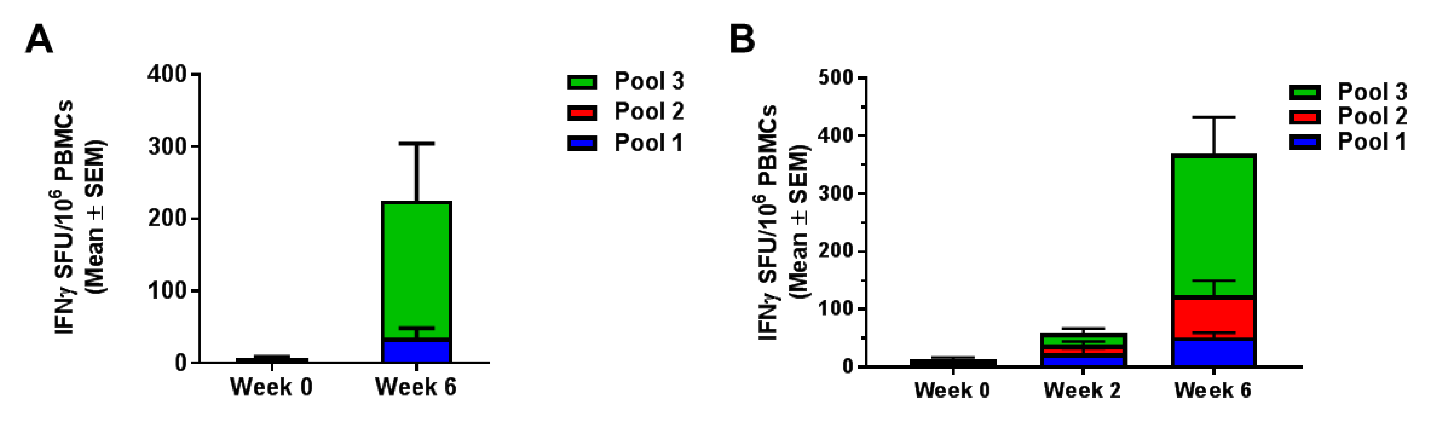


**Supplementary Figure S2. LASV peptide pool-specific cellular responses.** Specific cellular responses to individual LASV GPC peptide pools for IFNγ ELISpot assays depicted in Figure 5. (A) Pre-challenge LASV GPC peptide pool-specific cellular responses at the indicated timepoints post-immunization for pLASV-GPC DNA-immunized NHPs described in Figure 5A. (B) LASV GPC peptide pool-specific cellular responses at the indicated timepoints post pLASV-GPC immunization for NHPs in a follow-up study described in Figure 5B.
